# Contrast‐Enhanced Multispectral Optoacoustic Tomography for Functional Assessment of the Gastrointestinal Tract

**DOI:** 10.1002/advs.202302562

**Published:** 2023-06-08

**Authors:** Lars‐Philip Paulus, Adrian Buehler, Alexandra L. Wagner, Roman Raming, Jörg Jüngert, David Simon, Koray Tascilar, Alexander Schnell, Ulrich Rother, Markus Eckstein, Werner Lang, André Hoerning, Georg Schett, Markus F. Neurath, Maximilian J. Waldner, Regina Trollmann, Joachim Woelfle, Sarah E Bohndiek, Adrian P. Regensburger, Ferdinand Knieling

**Affiliations:** ^1^ Department of Pediatrics and Adolescent Medicine University Hospital Erlangen Friedrich‐Alexander‐Universität (FAU) Erlangen‐Nürnberg 91054 Erlangen Germany; ^2^ Pediatric Experimental and Translational Imaging Laboratory (PETI‐Lab) Department of Pediatrics and Adolescent Medicine University Hospital Erlangen Friedrich‐Alexander‐Universität (FAU) Erlangen‐Nürnberg 91054 Erlangen Germany; ^3^ Department of Pediatric Neurology, Center for Chronically Sick Children Charité Berlin Berlin Germany; ^4^ Department of Medicine 3, University Hospital Erlangen Friedrich‐Alexander‐Universität (FAU) Erlangen‐Nürnberg 91054 Erlangen Germany; ^5^ Department of Vascular Surgery University Hospital Erlangen Friedrich‐Alexander Universität (FAU) Erlangen‐Nürnberg 91054 Erlangen Germany; ^6^ Insitute of Pathology University Hospital Erlangen Friedrich‐Alexander‐Universität (FAU) Erlangen‐Nürnberg 91054 Erlangen Germany; ^7^ German Center Immunotherapy (DZI) University Hospital Erlangen Friedrich‐Alexander‐Universität (FAU) Erlangen‐Nürnberg 91054 Erlangen Germany; ^8^ Department of Medicine 1 University Hospital Erlangen Friedrich‐Alexander‐Universität (FAU) Erlangen‐Nürnberg 91054 Erlangen Germany; ^9^ Department of Physics University of Cambridge Cambridge CB3 0HE UK; ^10^ Cancer Research UK Cambridge Institute University of Cambridge Cambridge CB2 0RE UK

**Keywords:** indocyanine green, intestine, multispectral optoacoustic tomography, optoacoustics, photoacoustics

## Abstract

Real‐time imaging and functional assessment of the intestinal tract and its transit pose a significant challenge to conventional clinical diagnostic methods. Multispectral optoacoustic tomography (MSOT), a molecular‐sensitive imaging technology, offers the potential to visualize endogenous and exogenous chromophores in deep tissue. Herein, a novel approach using the orally administered clinical‐approved fluorescent dye indocyanine green (ICG) for bedside, non‐ionizing evaluation of gastrointestinal passage is presented. The authors are able to show the detectability and stability of ICG in phantom experiments. Furthermore, ten healthy subjects underwent MSOT imaging at multiple time points over eight hours after ingestion of a standardized meal with and without ICG. ICG signals can be visualized and quantified in different intestinal segments, while its excretion is confirmed by fluorescent imaging of stool samples. These findings indicate that contrast‐enhanced MSOT (CE‐MSOT) provides a translatable real‐time imaging approach for functional assessment of the gastrointestinal tract.

## Graphical abstract

Multispectral optoacoustic tomography (MSOT), a non‐invasive, molecular‐sensitive imaging technology, allows visualization and quantification of chromophores in deep tissue. In a translational approach, transabdominal contrast‐enhanced (CE) MSOT is performed after the oral intake of indocyanine green (ICG) for real‐time assessment of the gastrointestinal transit. CE‐MSOT offers the capabilities for immediate clinical translation to visualize numerous gastrointestinal pathologies.

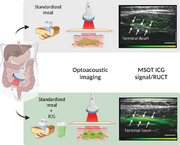

## 1 Introduction

Despite the availability of sophisticated cross‐sectional imaging techniques, anatomical and functional assessment of the gut – especially in children – is still frequently performed using X‐ray fluoroscopy.^[^ ^1^ ^]^ Such procedures require skilled interpretation, use ionizing radiation, and may have limited diagnostic information.^[^ ^2^ ^]^ In addition, these methods usually cannot provide information to dynamically monitor the passage of food, which would enable diagnosing a variety of functional intestinal diseases.

Alternatively, ultrasound imaging techniques offer high‐resolution, real‐time radiation‐free imaging capabilities.^[^ ^3^, ^4^ ^]^ Multispectral optoacoustic tomography (MSOT) combined with ultrasound can be seen as a further development in this field, which, in addition to anatomical B‐mode information, also enables molecular imaging of tissues.^[^ ^5^ ^]^ MSOT relies on the absorption of laser light in the near‐infrared window to induce a thermoelastic response, resulting in detectable ultrasound waves.^[^ ^6^ ^]^ This allows the visualization and quantification of different endogenous chromophores like hemoglobin, lipids, and collagens.^[^ ^6^, ^7^, ^8^, ^9^ ^]^ Label‐free clinical MSOT has already demonstrated feasibility for non‐invasive assessment of hemoglobin signals as surrogate markers for intestinal inflammatory activity.^[^ ^10^, ^11^ ^]^

To increase the specificity for detection, externally applied contrast agents with distinct optoacoustic spectra^[^ ^12^, ^13^ ^]^ can be used for precise optoacoustic visualization.^[^ ^14^, ^15^, ^16^ ^]^ While contrast‐enhanced (CE) imaging typically requires intravenous application and in optics, often near surface detection,^[^ ^17^, ^18^ ^]^ MSOT goes beyond these limitations and enables clinically relevant transabdominal deep tissue penetration.^[^ ^19^, ^20^, ^21^ ^]^

We hypothesized that the oral administration of indocyanine green (ICG), a clinical approved dye, might enable the visualization of gastrointestinal passage in a non‐invasive radiation‐free manner to close the gap in the current standard of care. To test this hypothesis, we performed phantom experiments and a clinical trial for immediate translation of this approach for clinical applications.

## 2 Results

### 2.1 ICG Imaging in Phantom

**Figure 1:**
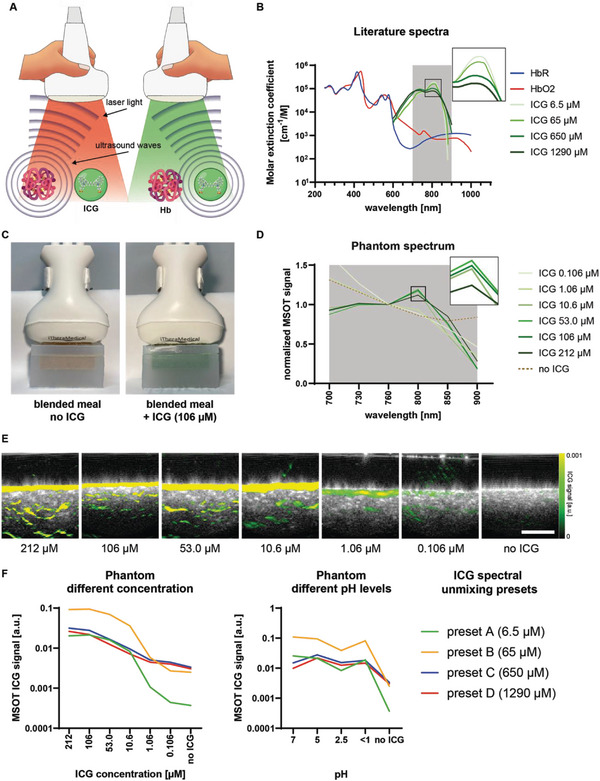
Multispectral optoacoustic imaging of ICG in phantoms. A) Handheld multispectral optoacoustic tomography emits laser light at different wavelengths allowing differentiation of various chromophores such as hemoglobin or indocyanine green (ICG). Hb = hemoglobin, ICG = indocyanine green. Image created with BioRender.com. Molecular structure of ICG derived from http://molview.org on 12/01/2022. B) Endogenous (hemoglobin) and exogenous (ICG) chromophores and their respective molar extinction coefficients in the near‐infrared range of light. All spectra derived from https://omlc.org last accessed 12/01/2022 and Landsman et al. ^[^ ^13^ ^]^ HbR = deoxygenated hemoglobin, HbO2 = oxygenated hemoglobin, ICG = indocyanine green. C) Agarose phantoms from 3D printed molds filled with standardized blended meals with and without ICG for MSOT imaging. D) Normalized optoacoustic spectra derived from blended meal phantoms with and without ICG (concentrations range between 0.106 and 212 µM). Absolute values are normalized on the 760 nm signals for each dataset. E) MSOT ICG signals detected in phantoms with blended meals with and without ICG (concentrations range between 0.106 and 212 µM). White bar represents 1 cm. F) MSOT ICG signals derived from different spectral unmixing settings (preset A–D) in phantoms with different ICG concentrations and pH levels.

For all experiments, a handheld clinically‐certified MSOT was used for ICG imaging (**Figure** **1**a). To differentiate ICG from background chromophores such as hemoglobin, spectral unmixing was applied using the unique absorption spectra of the molecules of interest. The absorption spectra of ICG are known to change slightly in a dose‐dependent manner, but all peak at around 800 nm (Figure 1b). In phantom experiments, blended meals with and without ICG at six different concentrations (0.106–212 µM) were examined for their respective optoacoustic spectra (Figure 1c,d). ICG concentrations ranging from 10.6–212 µM confirmed a signal maximum at 800 nm in accordance with the molecular extinction coefficient derived from the literature (Figure 1b,d). In anticipation of the clinical study, in which all subjects had a standardized meal with an ICG concentration of about 106 µM (50 mg ICG in 150 mL of water with a meal of ≈450 mL), we wanted to investigate the limit of detection for ICG in the phantom setting. Assuming the concentration decreases due to the large distribution volume in the gastrointestinal tract, ICG concentrations between 0 and 212 µM were tested. As demonstrated, all concentrations down to 0.106 µM were detected by MSOT while the negative control did not show any evidence of ICG (Figure 1e). To mimic gastric passage and its acid condition, we confirmed that changing pH levels had no relevant influence on the detection of ICG by MSOT (Figure 1f). With the clinical MSOT system, four ICG spectral unmixing settings were available for data analysis derived from the concentration‐dependent molar extinction spectra of ICG in plasma from Landsman et al.^[^ ^13^ ^]^ The use of preset A (using the spectrum derived from an ICG concentration of 6.5 µM) showed the best discrimination between different samples (Figure 1f). Furthermore, CE‐MSOT allowed the detection of ICG at different imaging depths (5–25 mm) in phantom experiments according to the depth variations of the human intestine (Figure S1, Supporting Information).

### 2.2 Clinical Proof‐of‐Concept Trial

**Figure 2:**
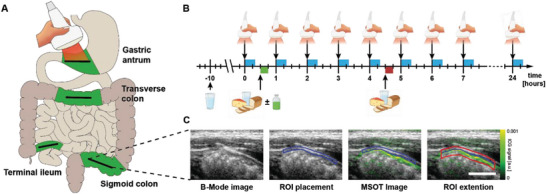
Study flow and MSOT data processing. A) Four intestinal segments (gastric antrum, terminal ileum, transverse colon, and sigmoid colon) were imaged by handheld MSOT. B) MSOT imaging was performed on eight timepoints with an interval of 60 minutes on two separate days. On both days subjects received two standardized meals 30 min after the first and fifth imaging time point. On the second day 50 mg of ICG in 150 ml water was ingested by every subject after the first meal. C) Co‐registered B‐mode images were used for anatomical guidance during region of interest (ROI) placement. Thereafter, a semi‐automated ROI extension was applied to outline the area of potential ICG signal detection. White bar represents 1 cm.

*n* = 10 healthy subjects (*n* = 6 females) were investigated within the clinical trial. The subjects had a mean ± SD age of 22.5 ± 1.2 years, height of 174.7 ± 9.1 cm, weight of 66.5 ± 7.8 kg, and BMI of 21.7 ± 1.1 kg m^−2^. Transabdominal MSOT imaging of four anatomical locations was performed consecutively at each of eight imaging time points every 60 min on 2 days with an interval of at least 48 h in‐between (**Figure** **2**a,b). All subjects had two standardized meals and on the second imaging day an additional oral intake of 50 mg ICG after the first meal. MSOT imaging was guided by hybrid B‐mode ultrasound to identify the intestinal segments. After image acquisition, ROI placement, semi‐automated ROI extension and subsequent batch mode analyses of MSOT ICG signals with different ICG spectral unmixing presets was performed (Figure 2c). The presented analyses were computed with an ICG unmixing preset A (derived from an ICG concentration of 6.5 µM in plasma). As described above, this showed the most distinguishable results in phantom experiments and might reflect the in vivo situation best with decreased ICG concentrations in the downstream intestinal segments. All analyses were similarly re‐performed with the other spectral unmixing presets, indicating similar results (see Figures S2–S4, Supporting Information).

### 2.3 Standard Assessments of Intestinal Transit

**Figure 3:**
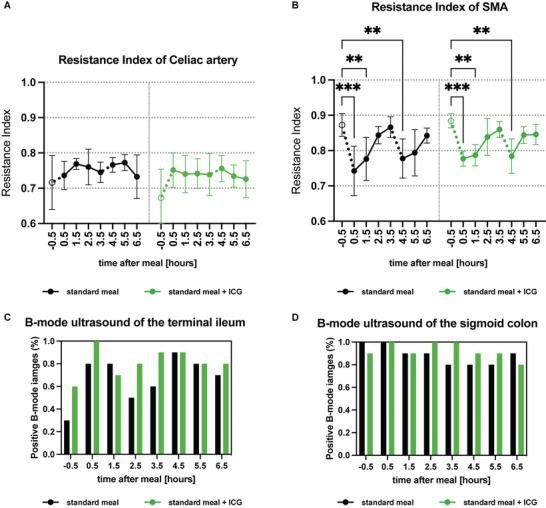
Standard assessments of gastrointestinal transit. A) Resistance Index of the celiac artery during the course of the clinical trial for both days (black = standard meal, green = standard meal + ICG). Dots and whiskers represent mean ± SD. B) Resistance index of the superior mesenteric artery during the course of the clinical trial for both days (black = standard meal, green = standard meal + ICG). Dots and whiskers represent mean ± SD. Asterisks represent significant differences. ** *p* < 0.01, *** *p* < 0.001. C) Scoring of presence of feces (yes or no) in the terminal ileum derived from B‐mode ultrasound images of all time points (black = standard meal, green = standard meal + ICG). Bars show percentages of positive presence of feces. D) Scoring of presence of feces (yes or no) in the sigmoid colon derived from B‐mode ultrasound images of all time points (black = standard meal, green = standard meal + ICG). Bars show percentages of positive presence of feces.

In order to test the validity of the approach, we assessed other standard methods to derive information from the intestinal passage. Ultrasound Doppler imaging showed no significant changes in the celiac artery, while the resistance index in the superior mesenteric artery significantly decreased both, after the standard meal and meal+ICG intake at 0.5 h (meal: *p* = 0.0001, meal+ICG: *p* = 0.0004), 1.5 h (*p* = 0.0076, *p* = 0.0022) and 4.5 h (*p* = 0.0020, *p* = 0.0034) as compared to −0.5 h (**Figure** **3**a,b).

Scoring of the presence or absence of feces in the intestinal lumen by RUCT images revealed a passage of feces into the terminal ileum. However, the sigmoid colon was filled with feces during all timepoints. (Figure 3c,d).

### 2.4 In Vivo Contrast‐Enhanced MSOT in Humans

**Figure 4:**
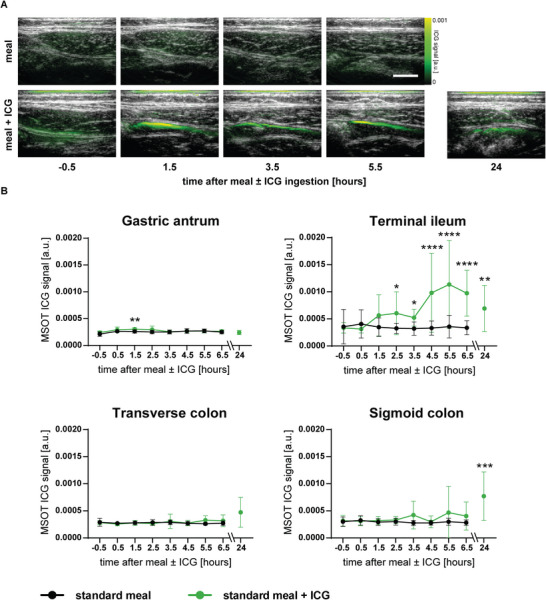
Contrast‐enhanced MSOT for the assessment of gastrointestinal transit. A) MSOT ICG signals were detected from 1.5 h after oral ICG intake in the terminal ileum. In contrast, no ICG signals were detected in the control study arm. White bars represent 1 cm. B) MSOT ICG signal quantification of each imaging time point of the day with and without ICG ingestion in the gastric antrum, terminal ileum, transverse colon, and sigmoid colon. Dots and whiskers represent mean ± SD. Asterisks represent significant differences. * *p* < 0.05, ** *p* < 0.01, *** *p* < 0.001, **** *p* < 0.0001.

At the beginning of the clinical study, no relevant ICG signals were seen throughout all observed intestinal segments. In the gastric antrum, ICG signals were increased after 1.5 h (25.4 ± 3.8 × 10^−5^ vs 30.5 ± 2.7 × 10^−5^ a.u., *p* = 0.0010). At this time point, the first signal increases were also detectable in the more distal terminal ileum (**Figure** **4**a and Video S1, Supporting Information). Herein, unmixed ICG signals were elevated over the time range from 2.5 to 24 h with a maximum after 5.5 h (34.8 ± 18.3 × 10^−5^ vs 113.5 ± 81.1 × 10^−5^ a.u., *p* < 0.0001). However, no significant differences in MSOT ICG signal intensity were found in the transverse colon. In the more distal sigmoid colon, there was an enrichment of the signal after 24 h (29.4 ± 6.4 × 10^−5^ vs 77.1 ± 44.7 × 10^−5^ a.u., *p* = 0.0008) (Figure 4b and Video S2, Supporting Information).

### 2.5 Proof of Successful Gastrointestinal Passage

**Figure 5:**
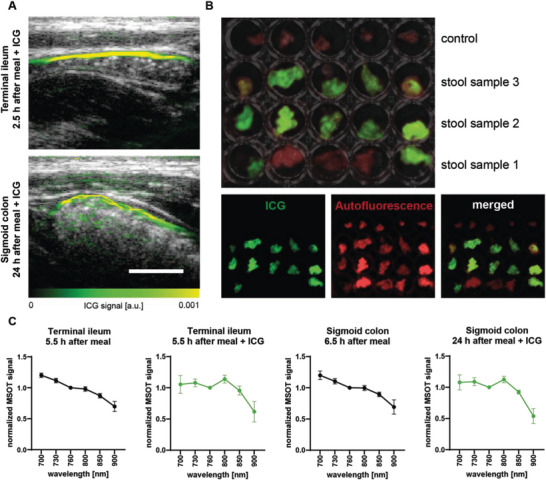
Confirmation of MSOT ICG signals in the stool. A) MSOT ICG signals were detected timely after oral ICG intake in the terminal ileum and with expected delay on the next day in the sigmoid colon. White bars represent 1 cm. B) From *n* = 5 subjects, three consecutive stool samples after oral ICG intake were analyzed by fluorescent imaging. Factory presets for ICG fluorescence and tissue autofluorescence signals were used. C) Optoacoustic spectra 5.5 h after ICG intake in the terminal ileum and 24 h after ICG intake in the sigmoid colon compared with corresponding time points of the day without ICG intake. Dots and whiskers represent mean ± SD.

As MSOT ICG signal increase in the colon was only seen 24 h after ICG intake, we wanted to confirm a successful passage of the compound through the gastrointestinal tract without systemic uptake or intestinal degradation (**Figure** **5**a). Therefore, fluorescent imaging was performed on three subsequent stool samples from *n* = 5 subjects (Figure 5b). The three stool samples were collected 16.7 ± 17.3, 30.6 ± 23.6, and 42.8 ± 30.6 h after ICG ingestion. Three out of five samples from the first time point and all further samples were positive for ICG fluorescence. To further confirm the presence of ICG, optoacoustic spectra were analyzed at the imaging time points with the greatest ICG signal intensities (5.5 h after ICG intake in the terminal ileum and 24 h after ICG intake in the sigmoid colon). These optoacoustic ICG spectra were found to be similar to the one derived from ICG phantom experiments (Figure 5 c, Figure 1d), while respective timepoints of the day without ICG intake showed different spectra (Figure 5c).

## 3 Discussion

In this translational study, we have demonstrated the technical and clinical feasibility of CE‐MSOT for functional imaging of the intestine. While phantom experiments showed that ICG detection is possible over a wide range of concentrations and pH conditions, a subsequent clinical trial confirmed the feasibility for in vivo imaging of the human intestine. Furthermore, fluorescent ICG signals were also detectable in stool samples after the oral intake of the dye.

ICG, a dye already approved for intravenous use in children, adolescents, and adults, has already shown broad applications in preclinical^[^ ^22^, ^23^, ^24^ ^]^ and clinical studies.^[^ ^25^, ^26^ ^]^ Similarly to the current study, Morscher et al. demonstrated its oral use for imaging gastric emptying in mice.^[^ ^27^ ^]^ Beyond these animal experiments, we demonstrated the feasibility of this drug/device combination for hand‐held, bedside imaging of the intestine with broad clinical implications. CE‐MSOT could allow for bedside assessment of the gastrointestinal passage and prolonged transit times in patients with chronic constipation without harmful ionizing or radioactive radiation of diagnostic standards like radio‐opaque marker testing^[^ ^28^, ^29^ ^]^ and scintigraphy.^[^ ^30^ ^]^ Furthermore, very young patients suspected of intestinal malformation, obstruction, functional bowel disorders, or fistulae^[^ ^31^, ^32^ ^]^ and chronically ill patients with strictures caused by inflammatory diseases^[^ ^33^ ^]^ could benefit from such a non‐invasive, radiation‐free molecular‐sensitive imaging approach, which could complement established magnetic resonance imaging or computed tomography protocols.^[^ ^34^ ^]^ The emission of ICG is within the near‐infrared band and can be detected from a surface depth of up to 4 mm by fluorescence imaging.^[^ ^35^, ^36^ ^]^ In comparison, CE‐MSOT provides depth penetration of around 1.5–2 cm to reliably detect the signal of ICG in deep tissue imaging.

While ultrasound Doppler signal measurements show only changes in blood flow and ultrasound B‐mode images show the presence of feces (whether new or old), CE‐MSOT can show a dynamic representation of the passage of bowel contents. In contrast to other studies, we used a clinical‐grade ICG compound and avoided the generation of any encapsulated formulations in order to potentially reduce the degradation of the contrast agent.^[^ ^37^ ^]^ Our phantoms experiments prove that an acid environment does not affect the optoacoustic properties of ICG and its stability within the gut was proven by its detection in stool samples. Future dyes would also need to remain stable during the entire intestinal passage, show as little enteral resorption as possible and ideally exhibit an absorption peak different from endogenous absorbers, such as hemoglobin. In this regard, also imaging at a higher wavelengths, the second near‐infrared window (1000 to 1700 nm), might provide a further road for development.^[^ ^38^ ^]^

In the future, contrast enhanced imaging may enable targeted imaging in the human intestine to directly flag inflammatory or malignant processes.^[^ ^39^ ^]^ In comparison to molecular endoscopy,^[^ ^17^, ^40^ ^]^ which uses similar principles, targeted CE‐MSOT with oral administration may avoid any invasive imaging procedures. However, prior targeted optoacoustic imaging studies using the intravenous application of either cetuximab‐ 800CW^[^ ^41^ ^]^ or panitumumab‐IRDye800CW^[^ ^42^ ^]^ for the detection of cancer metastasis showed heterogeneous results. The amounts of imaging agents given via systematic circulation might not have been sufficient to produce enough signal in comparison to background noise. This problem was not present in our study, wherein we used 50 mg of ICG, which is still significantly below the maximum recommended daily intravenous dose of 1.25 mg kg^−1^ per body weight. As ICG should not be absorbed during intestinal passage a systematic uptake of ICG in inflamed intestinal segments should not represent a relevant problem. In addition, oral as opposed to intravenous administration allowed us to circumvent the problem of possible misinterpretation of strong interfering hemoglobin signals in vessels. However, the influence of light absorption and optoacoustic signals arising from intestinal contents is unclear and should be investigated in further studies. In this study, we applied only a possible dosage and could not assess efficacy or pharmacokinetics. As our phantom data indicate, imaging signals might also be derived from significantly lower amounts of ICG (10.6 µm, ≈10%).

This study has several limitations. The reliable identification of anatomical structures is significantly more difficult with optoacoustic imaging, so a hybrid approach with B‐mode imaging was required to guide the examiner.^[^ ^43^, ^44^ ^]^ Imaging of signals in the gastric and transversal colon regions was not equal to the terminal ileum and sigmoid colon. In the stomach, no ICG enhancement was detectable after ICG ingestion even though an increased gastric filling was visible in the B‐mode image on time points directly after the meals. One reason might be the quick transit of liquids through gastric folds along the small curvature without proper mixing with solid food components.^[^ ^45^, ^46^ ^]^ For the transverse colon chyme retention time is most likely too short that the ICG could be imaged. Also, the distribution and dilution/concentration of ICG in the intestine at different anatomical locations is largely unknown. In this regard, we have shown that ICG was detectable from a concentration of 200% to 0.1% of the original meal with ICG (106 µM). Visually ICG signals within the intestine had the greatest similarity with signals from the phantom at 1.06 µM, indicating a rather low concentration in the gut.

In summary, the present translational study demonstrated the feasibility of MSOT for the detection of an orally applied,clinical approved dye inside the gastrointestinal tract. In contrast to other modalities, CE‐MSOT does not require any radiation and enables deep tissue detection. This suggests an immediate clinical translation in order to minimize the number of more invasive and burdensome examinations while opening the window for novel molecular imaging of gastrointestinal pathologies of functional and structural nature.

## 4 Experimental Section

### Phantom Experiments

For phantom experiments, custom‐made molds were designed using Autodesk Fusion 360 (V2.0.14567, Autodesk GmbH, Munich, Germany) and printed with a 3D printer (Form 2, Formlabs. Inc, Somerville, MA, USA) using White Resin V4 (Formlabs. Inc, Somerville, MA, USA). Two molds were filled with 2% agarose (Biozym LE Agarose, Biozym Scientific GmbH, Hessisch Oldendorf, Germany) dissolved in distilled water to cast an agarose phantom with the body dimension of 96 × 46 × 40 mm and a matching cover of 96 × 46 × 10 mm. Inside the body part, a 76 × 26 × 20 mm recess was used to be filled with different contents and sealed with the agarose cover. For the experiments, a standardized meal^[^ ^47^ ^]^ (≈450 mL, Table S1, Supporting Information) was blended and divided into 112.5 mL portions for each phantom setup. ICG (Verdye 5 mg mL^−1^, Diagnostic Green, Aschheim‐Dornach, Germany) was reconstituted according to the manufacturer's recommendations and different amounts of the solution were added to achieve the final concentrations in the blended meal samples. To test the stability of ICG signals at varying pH levels, one portion of the blended meal was acidified to a pH of 2.5 using 1.2 mL of 32% HCl before ICG was added. Another portion was similarly treated and adjusted to a pH of 7 using 1.6 mL of 32% NaOH. This should simulate the change of pH during the gastrointestinal transit from the stomach to the duodenum and small intestine. Furthermore, another portion was further acidified with 32% HCl to reach a pH below 1 (Table S2, Supporting Information). To demonstrate the detection of ICG at different imaging depths according to the depth variation of the intestine a custom‐made phantom with plastic straw placeholders at defined depths was used. Plastic straws were filled with ICG (5 mg ml^−1^) and imaged, accordingly.

For all experiments, the MSOT imaging probe (MSOT Acuity CE, iThera Medical, München, Germany) was mounted in a laboratory bracket and coupled to the agarose phantom using transparent ultrasound gel (Aquasonic Clear, MDSS GmbH, Hannover, Germany).

### Design and Flow of the Study

The prospective single‐center investigator‐initiated trial (IIT) was conducted from November 2021 to January 2022 at the Department of Pediatric and Adolescent Medicine at the University Hospital Erlangen. Approval from the local ethics committee was granted (346_21B) and the clinical trial was registered (clinicaltrials.gov identifier NCT05160077) before study initiation. The Declaration of Helsinki was respected and all subjects gave their written informed consent.

This study included *n* = 10 healthy subjects aged over 18 years. Exclusion criteria were pregnancy, nursing mothers, tattoos in the field of investigation, subcutaneous fat tissue over 3 cm, preexisting chronic or acute diseases of the gastrointestinal tract or symptoms suggestive of such a disease, diseases requiring acute treatments, and the lack of written consent. Furthermore, any known hypersensitivity to ICG, sodium iodide or iodine, hyperthyroidism, focal or diffuse thyroid autonomy, treatment with radioactive iodine for the diagnostic examination of thyroid function within 2 weeks before or after the study, and restricted renal functionand the following co‐medications also led to an exclusion: Beta‐blockers, anticonvulsants, cyclopropane, bisulfite compounds, haloperidol, heroin, meperidine, metamizole, methadone, morphine, nitrofurantoin, opium alkaloids, phenobarbital, phenylbutazone, probenecid, rifamycin, and any injection containing sodium bisulfite.

After inclusion, subjects were examined on two separate days, one with standardized meals, the other with standardized meals and oral ICG intake. For every day, two subjects were fasting since the evening before and started the examinations on the next day at 8:00 or 8:30 am, respectively. All subjects were imaged at *n* = 8 time points with an interval of 60 min in‐between and once 24 h after ICG intake. Before each imaging time point, subjects were advised to drink 150 mL of water, which reflected the recommended daily water amount of 1.500–2000 mL. 30 min after the first and fifth imaging time point, a standardized meal (500 kcal for women and 650 kcal for men: 55% carbohydrates, 29% fat, and 16% proteins^[^ ^47^ ^]^) was given. On the second day, 50 mg of ICG (Verdye 5 mg ml^−^, Diagnostic Green GmbH, Aschheim‐Dornach, Germany) dissolved in 150 mL water was orally administered after the first meal. Anatomical locations for imaging were as follows: gastric antrum, terminal ileum, transverse colon, and sigmoid colon. Two MSOT scans per location were performed at each time point.

### MSOT Device

The identical CE‐certified MSOT system (Acuity ECHO CE, iThera Medical GmbH, Munich, Germany) was used for experimental and clinical studies. This system enabled hybrid B‐mode (reflection ultrasound computed tomography, RUCT) and optoacoustic imaging.^[^ ^43^, ^44^ ^]^ All subjects were investigated with a handheld 2D detector and coupling was achieved by transparent ultrasound gel (Aquasonic Clear, MDSS GmbH, Hannover, Germany). All attending persons wore laser safety goggles during imaging. All anatomical regions were identified by B‐mode imaging and optoacoustic signals were acquired at 700, 730, 760, 800, 850, and 900 nm.

### Intestinal Passage Standard Assessments

As described elsewhere,^[^ ^48^ ^]^ a Siemens Acuson X300 (Siemens Medical Solutions Inc., Mountain View, USA) and a GE Logiq E9 (GE Healthcare, Wauwatosa, USA) were used for Doppler‐derived resistance index measurements of the celiac artery and the superior mesenteric artery (SMA).

In addition, RUCT images were used to assess the intestinal passage post hoc. RUCT images of each imaging timepoint were scored by two investigators for the presence or absence of stool in the intestinal lumen.

### Fluorescence Imaging

On the next day after ICG ingestion, *n* = 3 stool samples of consecutive timepoints were collected from *n* = 5 subjects. The fluorescent properties of these samples were examined using an in vivo imaging system (IVIS Spectrum, PerlkinElmer Inc., Waltham, MA, US) using excitation wavelengths of 570, 605, 640, 675, 710, and 745 nm and emission wavelengths of 760, 780, 800, 820, and 840 nm. To spectrally unmix for ICG signals in the stool samples, the factory presets for ICG fluorescence and tissue autofluorescence signals were used.

### Data Analysis of MSOT Scans

For analysis of phantom experiments, regions of interest (ROI) were drawn directly below the upper border of the recess in the agarose phantom to include the ICG signal using software supplied by the manufacturer (iLabs V 1.3.16, iThera medical GmbH, Munich, Germany). For all scans of the clinical study, ROIs resembling the upper bowel wall were drawn directly after image acquisition guided by the B‐mode image. The data were then transferred and processed with iLabs software (V 1.3.16, iThera medical GmbH, Munich, Germany) as follows: ROIs were checked by a second reader. After final placement, ROIs of the clinical study were automatically enlarged by 2 mm in depth using a custom‐written script to include the bowel lumen/stool as the potential area of ICG signals. This approach was used to ensure a similar analysis of scans with and without visible ICG signals. Within each ROI the mean of the highest 10% of pixels were used for batch mode quantification of single wavelengths and unmixed ICG signal levels. Spectral unmixing of ICG was performed using four different presets (A–D), each of which used a different reference spectrum for ICG in plasma, to account for the concentration‐dependent change in the ICG spectrum (reference spectra taken at 6.5, 65, 650, and 1290 µM). Spectral unmixing was applied to MSOT data acquired using the following wavelengths: 700, 730, 760, 800, and 850 nm.

### Statistics

Demographic data of all subjects were presented as numbers and percentages or mean and standard deviation. Data were tested for normal distribution by Shapiro–Wilk test. The MSOT spectra in phantom experiments were normalized to the value at 760 nm since at this wavelength the molar extinction coefficient of ICG is stable between concentrations. For clinical data, absolute values were used. For each imaging time point of the second day (meal + ICG), the ICG signal was tested against the mean of all time points of the first day (meal without ICG) using Kruskal–Wallis test with Dunn´s correction. Doppler data were compared between the time point before and every time point after the meals (with ICG) by repeated measures one‐way ANOVA or Friedmann test, as appropriate. *p*‐values < 0.05 indicated statistical significance. All statistical analyses were calculated by GraphPad Prism (Version 9, GraphPad Software Inc., La Jolla, USA).

## Conflict of Interest

M.J.W., F.K., and A.P.R. are shared patent holders together with iThera Medical on an optoacoustic imaging system/software described in the study.

## Author Contributions

A.P.R. and F.K. contributed equally to the work. L.P.P., A.P.R., and F.K. conceived the idea of the study. F.K. was the principal investigator. L.P.P., R.R., A.P.R., and F.K. performed preclinical and clinical imaging (MSOT and ultrasound). S.E.B. helped with phantom imaging design and spectral analysis. L.P.P., K.T., D.S., A.P.R., and F.K. performed (statistical) data analysis. L.P.P., A.L.W., A.P.B., R.R., J.J., D.S., K.T., A.S., U.R., M.E., W.L., A.H., G.S., M.F.N., J.W., M.J.W., F.K., and A.P.R. interpreted the data. L.P.P., A.P.R., and F.K. wrote the first draft of the manuscript. All other authors revised it critically for important intellectual content and approved the final version of the manuscript.

## Supporting information

Supporting Information

Supplemental Video 1

Supplemental Video 2

## Data Availability

The data that support the findings of this study are available on request from the corresponding author. The data are not publicly available due to privacy or ethical restrictions.

## References

[1] S. Kurugoglu , U. Korman , I. Adaletli , D. Selcuk , Pediatr. Radiol. 2007, 37, 457. 17377787 10.1007/s00247-007-0435-z

[2] O. J. Arthurs , M. J. Graves , A. D. Edwards , I. Joubert , P. A. k Set , D. J. Lomas , BMC Med. Imaging 2014, 14, 33. 25245815 10.1186/1471-2342-14-33 PMC4186814

[3] T. R. Sanchez , B. Doskocil , R. Stein‐Wexler , J. Ultrasound Med. 2015, 34, 59. 25542940 10.7863/ultra.34.1.59

[4] S. A. Anupindi , D. J. Podberesky , A. J. Towbin , J. Courtier , M. S. Gee , K. Darge , J. R. Dillman , Abdom. Imaging 2015, 40, 975. 25920487 10.1007/s00261-015-0423-y

[5] A. P. Regensburger , A. L. Wagner , J. Claussen , M. J. Waldner , F. Knieling , Mol. Cell. Pediatr. 2020, 7, 3. 32130546 10.1186/s40348-020-00095-4 PMC7056767

[6] V. Ntziachristos , D. Razansky , Chem. Rev. 2010, 110, 2783. 20387910 10.1021/cr9002566

[7] S. Tzoumas , N. Deliolanis , S. Morscher , V. Ntziachristos , IEEE Trans. Med. Imaging 2014, 33, 48. 24001986 10.1109/TMI.2013.2279994

[8] A. P. Regensburger , L. M. Fonteyne , J. Jüngert , A. L. Wagner , T. Gerhalter , A. M. Nagel , R. Heiss , F. Flenkenthaler , M. Qurashi , M. F. Neurath , N. Klymiuk , E. Kemter , T. Fröhlich , M. Uder , J. Woelfle , W. Rascher , R. Trollmann , E. Wolf , M. J. Waldner , F. Knieling , Nat. Med. 2019, 25, 1905. 31792454 10.1038/s41591-019-0669-y

[9] Q. Cao , N. G. Zhegalova , S. T. Wang , W. J. Akers , M. Y. Berezin , J. Biomed. Opt. 2013, 18, 101318. 23933967 10.1117/1.JBO.18.10.101318 PMC3739874

[10] M. J. Waldner , F. Knieling , C. Egger , S. Morscher , J. Claussen , M. Vetter , C. Kielisch , S. Fischer , L. Pfeifer , A. Hagel , R. S. Goertz , D. Wildner , R. Atreya , D. Strobel , M. F. Neurath , Gastroenterology 2016, 151, 238. 27269244 10.1053/j.gastro.2016.05.047

[11] F. Knieling , C. Neufert , A. Hartmann , J. Claussen , A. Urich , C. Egger , M. Vetter , S. Fischer , L. Pfeifer , A. Hagel , C. Kielisch , R. S. Görtz , D. Wildner , M. Engel , J. Röther , W. Uter , J. Siebler , R. Atreya , W. Rascher , D. Strobel , M. F. Neurath , M. J. Waldner , N. Engl. J. Med. 2017, 376, 1292. 28355498 10.1056/NEJMc1612455

[12] J. P. Fuenzalida Werner , Y. Huang , K. Mishra , R. Janowski , P. Vetschera , C. Heichler , A. Chmyrov , C. Neufert , D. Niessing , V. Ntziachristos , A. C. Stiel , Anal. Chem. 2020, 92, 10717. 32640156 10.1021/acs.analchem.0c01902

[13] M. L. Landsman , G. Kwant , G. A. Mook , W. G. Zijlstra , J. Appl. Physiol. 1976, 40, 575. 776922 10.1152/jappl.1976.40.4.575

[14] A. Buehler , E. Herzog , D. Razansky , V. Ntziachristos , Opt. Lett. 2010, 35, 2475. 20634868 10.1364/OL.35.002475

[15] N. Brillant , M. Elmasry , N. C. Burton , J. M. Rodriguez , J. W. Sharkey , S. Fenwick , H. Poptani , N. R. Kitteringham , C. E. Goldring , A. Kipar , B. K. Park , D. J. Antoine , Toxicol. Appl. Pharmacol. 2017, 332, 64. 28755860 10.1016/j.taap.2017.07.019

[16] N. C. Burton , M. Patel , S. Morscher , W. H. P. Driessen , J. Claussen , N. Beziere , T. Jetzfellner , A. Taruttis , D. Razansky , B. Bednar , V. Ntziachristos , NeuroImage 2013, 65, 522. 23026761 10.1016/j.neuroimage.2012.09.053

[17] J. H. Lee , T. D. Wang , Lancet Gastroenterol. Hepatol. 2016, 1, 147. 28404071 10.1016/S2468-1253(16)30027-9

[18] Q.‐Y. Chen , J.‐W. Xie , Q. Zhong , J.‐B. Wang , J.‐X. Lin , J. Lu , L.‐L. Cao , M. Lin , R.‐H. Tu , Z.‐N. Huang , J.‐L. Lin , H.‐L. Zheng , P. Li , C.‐H. Zheng , C.‐M. Huang , JAMA Surg. 2020, 155, 300. 32101269 10.1001/jamasurg.2019.6033

[19] Schellenberg, M. W. , H. K. Hunt , Photoacoustics 2018, 11, 14. 30073147 10.1016/j.pacs.2018.07.001 PMC6068331

[20] I. Steinberg , D. M. Huland , O. Vermesh , H. E. Frostig , W. S. Tummers , S. S. Gambhir , J. Photoacoust. 2019, 14, 77. 10.1016/j.pacs.2019.05.001 PMC6595011 31293884

[21] A. P. Regensburger , E. Brown , G. Krönke , M. J. Waldner , F. Knieling , Biomedicines 2021, 9, 483. 33924983 10.3390/biomedicines9050483 PMC8145174

[22] S. Bhatnagar , K. D. Verma , Y. Hu , E. Khera , A. Priluck , D. E. Smith , G. M. Thurber , Mol. Pharm. 2018, 15, 1746. 29696981 10.1021/acs.molpharmaceut.7b00994 PMC5941251

[23] A. O. Beringhs , S. Singh , T. Valdez , X. Lu , Sci. Rep. 2020, 10, 14003. 32814802 10.1038/s41598-020-71054-2 PMC7438515

[24] C. Kim , K. H. Song , F. Gao , L. V. Wang , Radiology 2010, 255, 442. 20413757 10.1148/radiol.10090281 PMC2858815

[25] I. Stoffels , S. Morscher , I. Helfrich , U. Hillen , J. Leyh , N. C. Burton , T. C. P. Sardella , J. Claussen , T. D. Poeppel , H. S. Bachmann , A. Roesch , K. Griewank , D. Schadendorf , M. Gunzer , J. Klode , Sci. Transl. Med. 2015, 7, 317ra199. 10.1126/scitranslmed.aad1278 26659573

[26] I. Stoffels , P. Jansen , M. Petri , L. Goerdt , T. J. Brinker , K. G. Griewank , T. D. Poeppel , D. Schadendorf , J. Klode , JAMA Network Open 2019, 2, 199020. 10.1001/jamanetworkopen.2019.9020 PMC6694392 31411710

[27] S. Morscher , W. H. P. Driessen , J. Claussen , N. C. Burton , J. Photoacoust. 2014, 2, 103. 10.1016/j.pacs.2014.06.001 PMC4244636 25431754

[28] J. M. Hinton , J. E. Lennard‐Jones , A. C. Young , Gut 1969, 10, 842. 5350110 10.1136/gut.10.10.842 PMC1552992

[29] Schindlbeck, N. E. , A. G. Klauser , S. A. Muller‐Lissner , Z. Gastroenterol. 1990, 28, 399. 2275262

[30] H. A. Ziessman , Curr. Treat. Options Gastroenterol. 2016, 14, 452. 27682148 10.1007/s11938-016-0108-9

[31] K. E. Applegate , D. D. T. Maglinte , Pediatr. Radiol. 2008, 38, 272. 10.1007/s00247-008-0754-8 18256816

[32] A. G. Carroll , R. G. Kavanagh , C. Ni Leidhin , N. M. Cullinan , L. P. Lavelle , D. E. Malone , Acad. Radiol. 2016, 23, 559. 26857524 10.1016/j.acra.2015.12.014

[33] F. Rieder , G. Latella , F. Magro , E. S. Yuksel , P. D. R. Higgins , A. Di Sabatino , J. R. De Bruyn , J. Rimola , J. Brito , D. Bettenworth , G. Van Assche , W. Bemelman , A. D'hoore , G. Pellino , A. U. Dignass , J Crohn's Colitis 2016, 10, 873. 26928961 10.1093/ecco-jcc/jjw055

[34] D. Bettenworth , F. Rieder , Fibrog. Tissue Repair 2014, 7, 5. 10.1186/1755-1536-7-5 PMC4230721 24678903

[35] S. Stolik , J. A. Delgado , A. Pérez , L. Anasagasti , J. Photochem. Photobiol., B 2000, 57, 90. 11154088 10.1016/s1011-1344(00)00082-8

[36] M. Leiloglou , M. S. Kedrzycki , V. Chalau , N. Chiarini , P. T. R. Thiruchelvam , D. J. Hadjiminas , K. R. Hogben , F. Rashid , R. Ramakrishnan , A. W. Darzi , D. R. Leff , D. S. Elson , Sci. Rep. 2022, 12, 8607. 35597783 10.1038/s41598-022-12504-x PMC9124184

[37] C. A. Wood , S. Han , C. S. Kim , Y. Wen , D. R. T. Sampaio , J. T. Harris , K. A. Homan , J. L. Swain , S. Y. Emelianov , A. K. Sood , J. R. Cook , K. V. Sokolov , R. R. Bouchard , Nat. Commun. 2021, 12, 5410. 34518530 10.1038/s41467-021-25452-3 PMC8438038

[38] P. K. Upputuri , M. Pramanik , J. Biomed. Opt. 2019, 24, 040901. 30968648 10.1117/1.JBO.24.4.040901 PMC6990072

[39] R. Wang , C. Song , A. Gao , Q. Liu , W. Guan , J. Mei , L. Ma , D. Cui , Acta Biomater. 2022, 143, 418. 35219867 10.1016/j.actbio.2022.02.031

[40] R. Atreya , H. Neumann , C. Neufert , M. J. Waldner , U. Billmeier , Y. Zopf , M. Willma , C. App , T. Münster , H. Kessler , S. Maas , B. Gebhardt , R. Heimke‐Brinck , E. Reuter , F. Dörje , T. T. Rau , W. Uter , T. D. Wang , R. Kiesslich , M. Vieth , E. Hannappel , M. F. Neurath , Nat. Med. 2014, 20, 313. 24562382 10.1038/nm.3462 PMC4479137

[41] J. Vonk , J. Kukacka , P. J. Steinkamp , J. G. De Wit , F. J. Voskuil , W. T. R. Hooghiemstra , M. Bader , D. Jüstel , V. Ntziachristos , G. M. Van Dam , M. J. H. Witjes , J. Photoacoust. 2022, 26, 100362. 10.1016/j.pacs.2022.100362 PMC9079001 35541024

[42] N. Nishio , N. S. Van Den Berg , B. A. Martin , S. Van Keulen , S. Fakurnejad , E. L. Rosenthal , K. E. Wilson , J. Nucl. Med. 2021, 62, 648. 33008927 10.2967/jnumed.120.245241 PMC8844260

[43] E. Mercep , G. Jeng , S. Morscher , P.‐C. Li , D. Razansky , IEEE Trans. Ultrason. Ferroelectr. Freq. Control 2015, 62, 1651. 26415127 10.1109/TUFFC.2015.007058

[44] E. Mercep , X. L. Dean‐Ben , D. Razansky , IEEE Trans. Med. Imaging 2017, 36, 2129. 28541198 10.1109/TMI.2017.2706200

[45] M. Grimm , E. Scholz , M. Koziolek , J.‐P. Kühn , W. Weitschies , Mol. Pharm. 2017, 14, 4262. 28930464 10.1021/acs.molpharmaceut.7b00623

[46] A. Pal , J. G. Brasseur , B. Abrahamsson , J. Biomech. 2007, 40, 1202. 16934271 10.1016/j.jbiomech.2006.06.006

[47] R. S. Goertz , C. Egger , M. F. Neurath , D. Strobel , Ultraschall Med. 2012, 33, 380. 22723037 10.1055/s-0032-1312816

[48] L. P. Paulus , A. L. Wagner , A. Buehler , R. Raming , J. Jüngert , D. Simon , K. Tascilar , A. Schnell , J. Günther , U. Rother , W. Lang , A. Hoerning , G. Schett , M. F. Neurath , J. Woelfle , M. J. Waldner , F. Knieling , A. P. Regensburger , J. Photoacoust. 2023, 30, 100457. 10.1016/j.pacs.2023.100457 PMC9942118 36824387

