# Supplementary material for: Contrast‐Enhanced Multispectral Optoacoustic Tomography for Functional Assessment of the Gastrointestinal Tract
Source: Adv Sci (Weinh). 2023 Jun 8;10(23):2302562. doi: 10.1002/advs.202302562 (PMC10427354; doi:10.1002/advs.202302562)
Supplement: Supplementary file 1 — Supporting Information [file ADVS-10-2302562-s003.pdf]

ADVANCED SCIENCE
Open Access

Supporting Information

for *Adv. Sci.*, DOI 10.1002/advs.202302562

Contrast-Enhanced Multispectral Optoacoustic Tomography for Functional Assessment of the Gastrointestinal Tract

Lars-Philip Paulus, Adrian Buehler, Alexandra L. Wagner, Roman Raming, Jörg Jüngert, David Simon, Koray Tascilar, Alexander Schnell, Ulrich Rother, Markus Eckstein, Werner Lang, André Hoerning, Georg Schett, Markus F. Neurath, Maximilian J. Waldner, Regina Trollmann, Joachim Woelfle, Sarah E Bohndiek, Adrian P. Regensburger and Ferdinand Knieling**

Supplementary Appendix

The present supplementary appendix should give additional information to the readers of Paulus L.P., et al. **Contrast-enhanced Multispectral Optoacoustic Tomography for Functional Assessment of the Gastrointestinal Tract**

TABLE OF CONTENTS

Supplementary Tables **3**

Supplementary Table 1 – Composition of standardized meal *3*

Supplementary Table 2 – Phantom experimental setup *4*

Supplementary Figures **5**

Supplementary Figure 1 – Contrast-enhanced MSOT for the detection of ICG at different imaging depths *5*

Supplementary Figure 2 – in vivo Contrast-enhanced MSOT signals using preset B (65µM) *6*

Supplementary Figure 3 – in vivo Contrast-enhanced MSOT signals using preset C (650µM) *7*

Supplementary Figure 4 – in vivo Contrast-enhanced MSOT signals using preset D (1290µM) *8*

Supplementary References **9**

Supplementary Tables

Supplementary Table 1 – Composition of standardized meal

**Woman**	**Man**
**~ 60 kg**	**~ 80 kg**
500 kcal	650 kcal
1½ multigrain bread roll<br>1 portion of jam<br>½ cream cheese, lean	
½ portion of butter<br>1 slice of cheese	1 portion of butter<br>1 slice of cheese
< 65 kg:<br>up to 2x 200 ml water<br>>65 kg, < 70kg:<br>100 ml orange juice<br>up to 2x 200 ml water<br>>70 kg:<br>200 ml orange juice<br>up to 2x 200 ml water	<85 kg:<br>200 ml orange juice<br>up to 2x 200 ml water<br>>85 kg:<br>300ml orange juice<br>up to 2x 200 ml water

Supplementary Table 1 – Composition of standardized meal

Table from Paulus et al.[1]; The standardized meal[2] was adjusted to the gender and body weight of the subject. The energy content of approximately 400-700 kcal (8.2 kcal/kg) accounts for about 25% of the daily energy requirement. The nutrient composition is 55% carbohydrates, 29% fat and 16% proteins. For phantom experiments the average standardized meal for women with 100 ml orange juice and 200 ml water was used.

Supplementary Table 2 – Phantom experimental setup

phantom ID	ICG concentration	blended meal	ICG amount	H_20	additional acid / base	pH
1	212 µM	112.5 ml	25 mg	37.5 ml		5
2	106 µM	112.5 ml	12.5 mg	37.5 ml		5
3	53.0 µM	112.5 ml	6.25 mg	37.5 ml		5
4	10.6 µM	112.5 ml	1.25 mg	37.5 ml		5
5	1.06 µM	112.5 ml	125 µg	37.5 ml		5
6	0.106 µM	112.5 ml	12.5 µg	37.5 ml		5
7	0 µM	112.5 ml	0 µg	37.5 ml		5
8	106 µM	112.5 ml	12.5 mg	37.5 ml	+ 1.2 ml HCl (32%)	2.5
9	106 µM	112.5 ml	12.5 mg	37.5 ml	+ 1.2 ml HCl (32%)<br>+ 2.5 ml NaOH (32%)	7
10	106 µM	112.5 ml	12.5 mg	37.5 ml	+ access HCl (32%)	<1

Supplementary Table 2 – Phantom experimental setup

Overview of the phantom experiments and their respective composition.

Supplementary Figures

Supplementary Figure 1 – Contrast-enhanced MSOT for the detection of ICG at different imaging depths

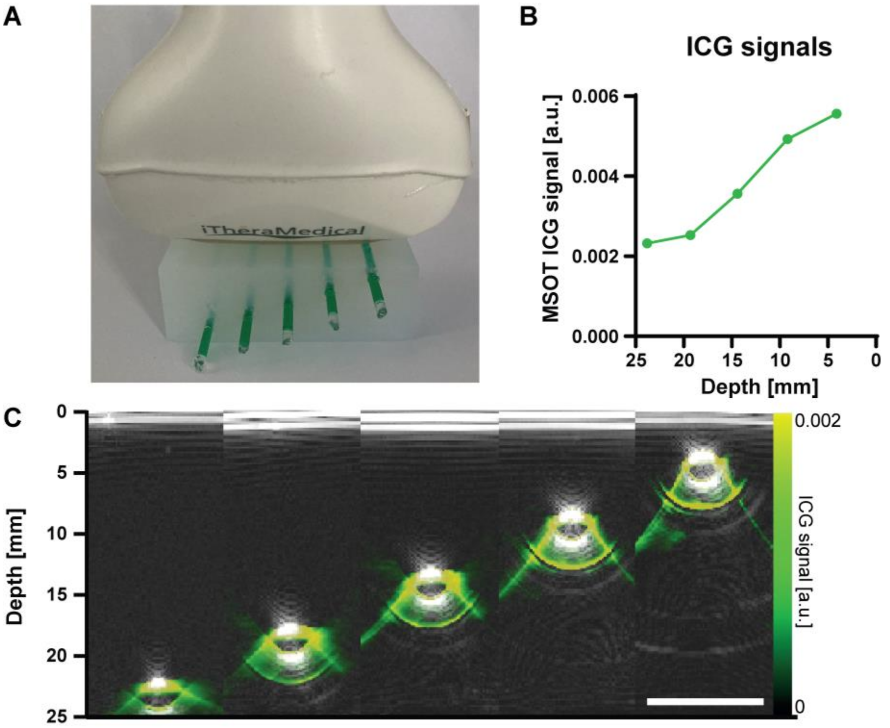

Supplementary Figure 1 – contrast-enhanced MSOT for the detection of ICG at different imaging depths

A: Agarose phantoms from 3D printed molds with defined imaging depths. Straws were filled with ICG (0.33 mg/ml) for MSOT imaging.
B: Quantification of MSOT ICG signal intensities at each imaging depth. MSOT ICG signals in arbitrary units (a.u.).
C: Detection and visualization of MSOT ICG signals at each imaging depth. Composite image of 5 MSOT scans. White bar indicates 1 cm.

Supplementary Figure 2 – *in vivo* Contrast-enhanced MSOT signals using preset B (65µM)

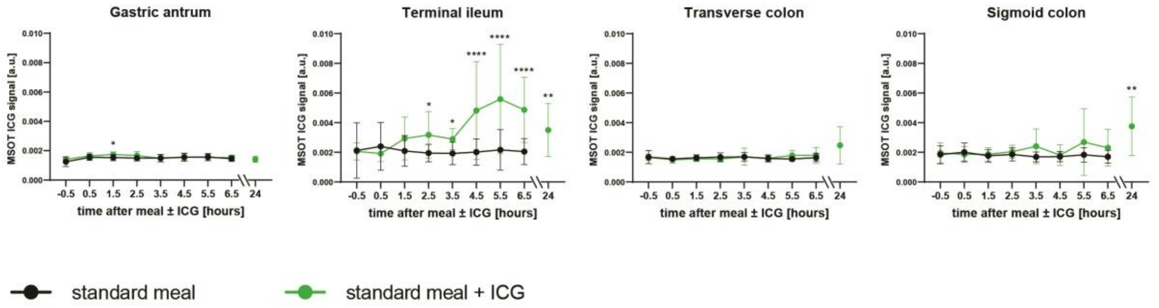

Supplementary Figure 2 – *in vivo* Contrast-enhanced MSOT signals using preset B (65µM)

MSOT ICG signal quantification of each imaging timepoint of the day with and without ICG ingestion in the gastric antrum, terminal ileum, transverse colon, and sigmoid colon. Dots and whiskers represent mean and SD. Asterikses represent significant differences. * *P*<0.05 ** *P*<0.01 *** *P*<0.001 **** *P*<0.0001.

Supplementary Figure 3 – *in vivo* Contrast-enhanced MSOT signals using preset C

(650µM)

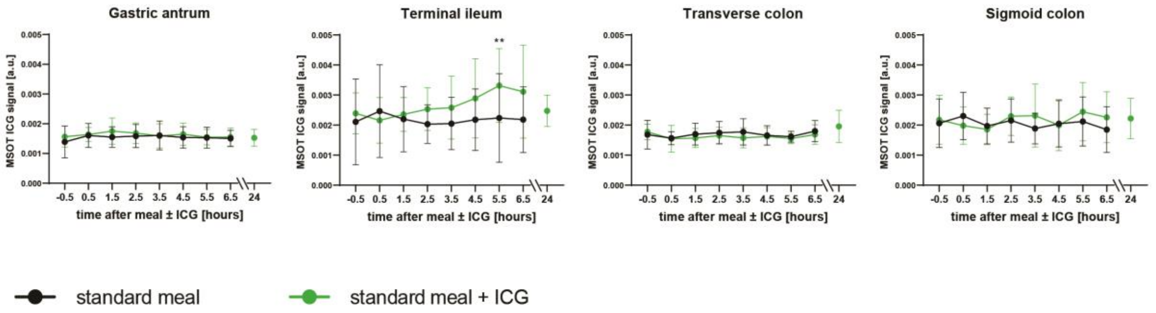

Supplementary Figure 3 – *in vivo* Contrast-enhanced MSOT signals using preset C (650µM)

MSOT ICG signal quantification of each imaging timepoint of the day with and without ICG ingestion in the gastric antrum, terminal ileum, transverse colon, and sigmoid colon. Dots and whiskers represent mean and SD. Asterikses represent significant differences. ** $P<0.01$.

Supplementary Figure 4 – *in vivo* Contrast-enhanced MSOT signals using preset D

(1290µM)

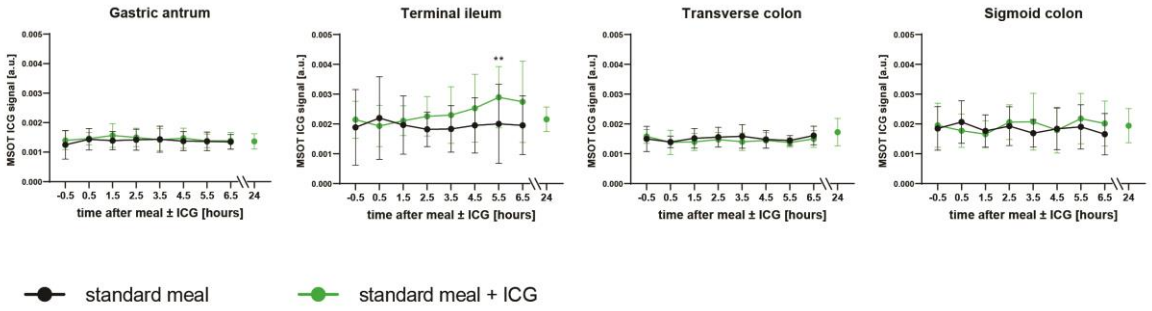

Supplementary Figure 4 – *in vivo* Contrast-enhanced MSOT signals using preset D (1290µM)

MSOT ICG signal quantification of each imaging timepoint of the day with and without ICG ingestion in the gastric antrum, terminal ileum, transverse colon, and sigmoid colon. Dots and whiskers represent mean and SD. Asterikses represent significant differences. ** *P*<0.01.

Supplementary References

1 Paulus, L. P. *et al.* Multispectral optoacoustic tomography of the human intestine - temporal precision and the influence of postprandial gastrointestinal blood flow. *Photoacoustics* **30**, 100457, doi:10.1016/j.pacs.2023.100457 (2023).

2 Goertz, R. S., Egger, C., Neurath, M. F. & Strobel, D. Impact of food intake, ultrasound transducer, breathing maneuvers and body position on acoustic radiation force impulse (ARFI) elastometry of the liver. *Ultraschall Med* **33**, 380-385, doi:10.1055/s-0032-1312816 (2012).
